# Spatiotemporal Propagation of the Cortical Atrophy: Population and Individual Patterns

**DOI:** 10.3389/fneur.2018.00235

**Published:** 2018-05-04

**Authors:** Igor Koval, Jean-Baptiste Schiratti, Alexandre Routier, Michael Bacci, Olivier Colliot, Stéphanie Allassonnière, Stanley Durrleman

**Affiliations:** ^1^Inria Paris-Rocquencourt, INSERM U1127, CNRS UMR 7225, Sorbonne Universités, UPMC Univ Paris 06 UMRS 1127, Institut du Cerveau et de la Moelle épinière, ICM, Paris, France; ^2^INSERM UMRS 1138, Centre de Recherche des Cordeliers, Université Paris Descartes, Paris, France; ^3^AP-HP, Pitié-Salpétriere Hospital, Department of Neurology, Paris, France; ^4^AP-HP, Pitié-Salpétriere Hospital, Department of Neuroradiology, Paris, France

**Keywords:** Alzheimer’s disease, cortical atrophy, brain networks, spatiotemporal propagation patterns, individual variability

## Abstract

Repeated failures in clinical trials for Alzheimer’s disease (AD) have raised a strong interest for the prodromal phase of the disease. A better understanding of the brain alterations during this early phase is crucial to diagnose patients sooner, to estimate an accurate disease stage, and to give a reliable prognosis. According to recent evidence, structural alterations in the brain are likely to be sensitive markers of the disease progression. Neuronal loss translates in specific spatiotemporal patterns of cortical atrophy, starting in the enthorinal cortex and spreading over other cortical regions according to specific propagation pathways. We developed a digital model of the cortical atrophy in the left hemisphere from prodromal to diseased phases, which is built on the temporal alignment and combination of several short-term observation data to reconstruct the long-term history of the disease. The model not only provides a description of the spatiotemporal patterns of cortical atrophy at the group level but also shows the variability of these patterns at the individual level in terms of difference in propagation pathways, speed of propagation, and age at propagation onset. Longitudinal MRI datasets of patients with mild cognitive impairments who converted to AD are used to reconstruct the cortical atrophy propagation across all disease stages. Each observation is considered as a signal spatially distributed on a network, such as the cortical mesh, each cortex location being associated to a node. We consider how the temporal profile of the signal varies across the network nodes. We introduce a statistical mixed-effect model to describe the evolution of the cortex alterations. To ensure a spatiotemporal smooth propagation of the alterations, we introduce a constrain on the propagation signal in the model such that neighboring nodes have similar profiles of the signal changes. Our generative model enables the reconstruction of personalized patterns of the neurodegenerative spread, providing a way to estimate disease progression stages and predict the age at which the disease will be diagnosed. The model shows that, for instance, APOE carriers have a significantly higher pace of cortical atrophy but not earlier atrophy onset.

## 1 Introduction

Neuroimaging studies have shown an alteration of the brain structure during the course of Alzheimer’s disease (AD) (1, 2). These lesions appear during the prodromal phase of the disease (3–5) whose observation have been limited due to the absence of clinical symptoms and diagnosis. The importance of the structural changes before the clinical symptoms led to hypothetical models (6), which have been later refined thanks to the gathering of multiple scientific evidences. These modifications took the form of a structural change of the brain in particular an important neuronal loss and an atrophy of the brain cortex (7, 8). The study of the temporal evolution of the cerebral cortex reveals an atrophy of the gray matter (9). This cortical atrophy presumably relates the traces of the progression of the lesions over the brain surface. A fine-scale modeling of the atrophy propagation is likely to give a wider understanding of the disease evolution, as the structural markers seems reliable to assess the conversion to the AD stage, potentially carrying subtle indicators of the disease progression in early phases.

The spatiotemporal propagation of these alterations encloses two entangled components. On the one hand, the spatial characterization of the lesions over the brain surface at each time, and, on the other hand, a temporal dynamic of these alterations that may differ from one region to another. Characterizing the proper dynamics of these lesions relies on the possibility to reconstruct the whole time-line of AD, at both a spatial and temporal level, out of short-term observations that are not temporally aligned. Another challenging aspect consists in the variability inherent to the individual patterns of atrophy that requires to consistently compare the subject-specific spreads of alterations. Accounting for the interindividual variability in term of lesion propagation should allow to reconstruct individual patterns of propagation, paving the way to possible personalized model of atrophy, that potentially informs on subject-specific age of conversion or disease stage.

Recently, large datasets have opened the opportunity to investigate data-driven models that have refined and validated these hypotheses to some extend, in particular event-based models (10–12) that considers the propagation as a series of events, allowing to define a sequence of disease stages. They characterize the overall variability of the events ordering at a population level. However, these models are not well suited to relate for the temporal delays of the alterations at a population level, neither to determine individual cortical atrophy. Multimodal observations, including positron emission tomography (PET) scans, magnetic resonance imaging (MRI) and biomarkers, have been gathered within longitudinal databases, i.e., repeated observations of patients during significant periods of time. The underlying intention is to provide multiple individual snapshots of the disease—patients examined during short-term periods—in order to reconstruct the long-term history of the pathology (13, 14) at a group and individual level. Moreover, it offers the possibility to describe and interpret the observed data contrary to quantiles or percentiles that require arbitrary reference distributions. A challenging aspect of AD patient comparison is the fact that, even though AD is related to age, the latter is not a good proxy of the disease stage (15–17) leaving us without any easy way to align all the individual on the same time-line. In Ref. (18), the authors introduced mixed-effect model that consider each individual trajectory as a variation of a mean scenario of evolution, with a time-warp function that is able to realign the subjects on the same time-line (19). It allows to characterize a spatial and temporal variability of propagation in the sense that it defines a group-average trajectory of propagation with the possibility to reconstruct individual observations thanks to personalized parameters. Nevertheless (18), constrain the model to parallel profiles of progression which does not hold when looking at signals that have various dynamics. Moreover, the model does not take into account the spatial correlations between the data whereas (17), which focus on spatiotemporal patterns of progression for images, exhibited that this led, in the case of a non-linear mixed-effects model, to poor estimations of the subject-specific parameters and individual trajectories.

To account for the spatial structure of the signal, networks have been introduced (20, 21), representing the brain areas as the graph nodes. In this paper, the networks correspond to a graph representation of a signal spatially distributed, namely, the cortical thickness mapped on a mesh representation of the cortex. The node values are the cortical thickness values over time on the related brain area. Extracting and projecting patients cortical thickness on the common mesh allows to compare their atrophy on the same atlas to exhibit similar patterns. As we expect the signal propagation to be spatially smooth with a similar temporal profile of change for neighbor nodes, we consider that a subset of the graph nodes act as control nodes. They define an evaluation function such that the signal at each node is an interpolation of the signal at the control nodes, enabling to smooth the high frequencies (22). The proximity between nodes is defined by the distance matrix which informs on the distance between any pair. Moreover, the number of nodes of this vertex-based graph can be tuned based on the desired application, potentially the same as the resolution of the input data, e.g., a voxel for MRI or PET data.

The aim of this paper is to introduce a model of the cortical atrophy propagation during the long-term course of AD thanks to a graph representation of the neuroimaging data. This model is able to personalize the reconstruction of the propagation to individual longitudinal measurements, allowing to describe the stages of the disease, potentially in the future. The model is described as a general framework for any longitudinal data spatially distributed on a common graph and it is instantiated to exhibit the propagation of the cortical atrophy on the left hemisphere of the brain, across nearly 2000 regions, thanks to longitudinal observations of 154 mild cognitive impaired (MCI) patients that were later diagnosed with AD. While exhibiting an average pattern of propagation, this mixed-effects model allows to reconstruct individual observations through time.

## 2 Materials and Methods

### 2.1 Sketch of the Method

**Figure 1:**
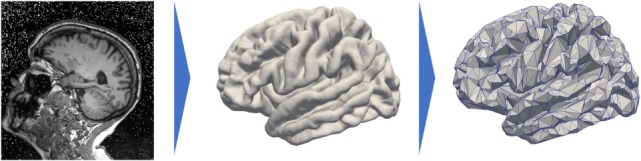
Data preprocessing that projects the cortical thickness of the raw MRI observation (left) on a mesh, namely, the FSAverage atlas constituted of 163.842 nodes per hemisphere (middle) before subsampling it and averaging the signal onto a 1,827-node graph (right).

Prior to detail our method, we would like to sketch the key ideas and notations of our work to ease and guide the reading. First, we consider *I* patients; each patient *i* is observed *J_i_* times, his *j*th visit being at age *t_ij_*, and each observation led to an MRI scan as shown on the left hand side of Figure 1. Segmentation of the cortical thickness, out of the neuroimaging observations, are mapped onto a mesh, as presented on the middle part of Figure 1. The last step corresponds to a subsampling process that led to a graph *G* of K nodes, characterized by a distance matrix *D*. At each node, the individual observations define a time-series describing the evolution of the signal through time.

**Figure 2:**
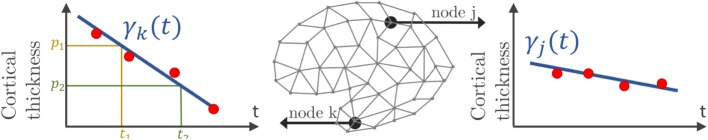
Mesh of the cortical surface where each node embeds a time-series of observations (red points). At node *k*, the function *γ_k_*(*t*), which can be parametrized by a velocity and two different sets (*p*_1_, *t*_1_) or (*p*_2_, *t*_2_), estimates the cortical thickness over time.

**Figure 3:**
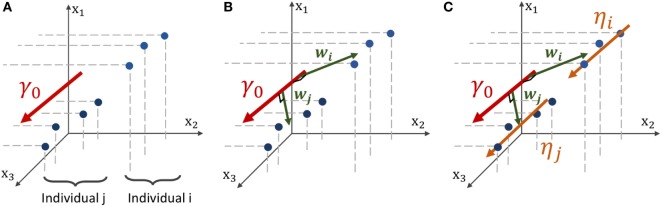
Geometric description of the construction of the mean and individual spatiotemporal trajectories in the space of measurement, which is the Riemannian Manifold *M* that embeds both the real observations and the trajectories. **(A)** Three-dimensional space embedding individual observations (blue points) of two individuals and the mean spatiotemporal trajectory *γ*_0_ (red curve). **(B)** The spatial variations from the group-average trajectory *γ*_0_ to the individual observations are captured in individual vectors *w_i_* and *w_j_*, called space shifts. **(C)** The vector *w_i_* is parallel-transported along *γ*_0_ (orange vectors) to define a parallel curve *η_i_* that characterizes the individual spatiotemporal trajectory.

In a second time, we assume that, at each node *k* of the graph *G*, there exists a function *t* ↦ *γ_k_*(*t*) that describes a characteristic evolution of the signal at this node, as shown on Figure 2. The time-series of individual *i* at node *k* derives from a continuous function *η_ik_*(*t*), which is assumed to be a spatial and temporal variations of the representative trajectory *γ_k_*(*t*), illustrated on Figure 3. The temporal variation corresponds to the time realignment of individual *i* on the common time-line. It adjusts the individual dynamics to a mean pace of evolution, thanks to personalized parameters *τ_i_* and *α_i_*. *τ_i_* stands for the individual time-shift to the mean disease onset, allowing an early (*τ_i_* < 0) or delayed (*τ_i_* < 0) age at diagnosis. The parameter *α_i_* integrates the patient-specific possibility to have a faster (*α_i_* > 1) or slower (*α_I_* < 1) pace of atrophy compared to the mean scenario of changes. On the other side, the spatial variation corresponds to the adjustment from the mean cortical thickness to individual data. It accounts, for instance, for the difference in size or in spatial thickness distribution at the same disease stage.

We consider that the characteristic signal *γ_k_*(*t*) at node *k* belongs to a family of curve, here the straight line curves, parametrized by the cortical thickness *p_k_* and the rate of atrophy *v_k_*. To account for the spatial structure of the signal and the large number of nodes, a subset of nodes, referred to as control nodes, is selected to control the interpolation of the cortical and atrophy values over all the nodes. The distribution of the control nodes depends on the size of the kernel bandwidth such that the kernels densities map almost uniformly the feature space.

The model introduces population parameters, that allow to define a characteristic spatiotemporal trajectory of the atrophy, and individual parameters, that not only enable to reconstruct individual trajectories but also permit the statistical study of the distribution of spatiotemporal atrophy patterns. These parameters are estimated thanks to the Monte-Carlo Markov-Chain Stochastic Approximation Expectation-Maximization (MCMC-SAEM) algorithm, which handles non-linear mixed-effects models, with theoretical guarantees and consistent results in practice.

### 2.2 Subjects and Data Preprocessing

Data used in the preparation of this article were obtained from the Alzheimer’s Disease Neuroimaging Initiative (ADNI) database (adni.loni.usc.edu). The ADNI was launched in 2003 as a public–private partnership, led by Principal Investigator Michael W. Weiner, MD. The primary goal of ADNI has been to test whether serial magnetic resonance imaging (MRI), positron emission tomography (PET), other biological markers, and clinical and neuropsychological assessment can be combined to measure the progression of mild cognitive impairment (MCI) and early Alzheimer’s disease (AD). For up-to-date information, see www.adni-info.org.

The Alzheimer’s Disease Neuroimaging Initiative (ADNI) dataset contains longitudinal MRI data for patients that are, at each visit, either cognitively normal (CN), mild cognitive impaired (MCI) patients, or, AD subjects. We selected all the subjects that presented a monotonous decline from MCI to AD, called the MCI converters, removing those that may convert from AD back to MCI or CN. Although AD patients get through an MCI phase, we could not keep CN to MCI patients as they might just as well convert to another dementia. Also, the patients that underwent from CN to MCI and then to AD are not numerous enough to give robust estimation of early stages (CN to MCI). Thus, we kept only the MCI to AD visits of such patients. Altogether, the paper focuses on 154 MCI patients that represents 787 visits, each individual being examined 5 times on average, from 2 to 7 times.

Each visit led to a T1-weighted MRI acquisition, as shown on the left side of Figure 1. The longitudinal pipeline of FreeSurfer (23) was used to extract the cortical thickness of the left hemisphere of the brain which was then projected on a common atlas, namely, FSAverage (24), which is a three-dimensional mesh composed of 163,842 nodes for each hemisphere represented on the central part of Figure 1. This common fixed-graph allows to compare the cortical thickness between visits or patients, node to node.

The data acquisition and interindividual alignment led to a considerable noise, especially in terms of variability in the measures for close nodes. To smooth this noise and to reduce the computational time, we subsampled the initial graph into a new graph of 1,827 nodes. To do so, we selected 1,827 nodes uniformly distributed over the whole FSAverage graph; the other nodes were then associated to one of the 1,827 nodes thanks to a geodesic distance *d* on the graph (i.e., the length of the shortest path on the surface mesh between the nodes) using the Fast Marching Algorithm on the mesh (25). Therefore, it constitutes collection of nodes referred to as patches. The value of each node of the subsampled graph is the average value over the corresponding patch, each being constituted of approximately 89 initial nodes of the FSAverage graph. The resolution of this vertex-based approach is lower than the initial one, shown on the right hand side of Figure 1, but still holds the brain topology while smoothing part of the acquisition noise. In our case, each observation can be considered as a vector of size 1,827 where the *k*th coordinate is related to the *k*th node of the common fixed-graph *G*. The latter is also described by the distance matrix *D* between the 1,827 nodes. It was obtained using the geodesic distance *d* between the 1,827 nodes on the initial graph FSAverage, whose edges are weighted by a physical length. Finally, for all *i*, *j* ∈ {1, … , 1,827}, we set *D_ij_* = d(**x***_i_*, **x***_j_*) where **x***_i_* and **x***_j_* are two nodes of the graph.

In the following, we will present a data-driven model which allow to track the propagation of any signal spatially distributed, supposedly the cortical thickness. We consider a longitudinal dataset **y** = (**y***_i,j_*)_1 ≤ *i* ≤ *I*, 1 ≤ *j* ≤ *Ji*_ of *I* individuals, each patient *i* being observed *J_i_* times during the study at ages (*t_ij_*)_1 ≤ *j* ≤ *Ji*_. We suppose that there exist a common fixed-graph *G* defined by a set $V=(x_{1},\ldots ,x_{K})$ of K nodes and a distance matrix *D* which accounts for the distance between the nodes. Any node $x_{k}\in R^{3}$ corresponds to a coordinate of a point in space. Each observation **y***_ij_* = = (**y***_ij_*_1_, … ,**y***_ijK_*) ∈ $R^{k}$ corresponds to the measured signal spatially distributed over the N nodes of *G*, represented by a point in the multivariate space $R^{k}$, schematically represented on Figure 3A for *K* = 3, as if there were only 3 vertices in the mesh. Therefore, it defines a network whose nodes are valued with the signal of interest. It follows that the collection (**y***_ij_*)_1 ≤ *j* ≤ *Ji*_ of the observations of a particular subject defines a network that embeds a time-series on each node of *G*, indexed by the patient age at each observation (*t_ij_*)_1 ≤ *j* ≤ *Ji*_.

### 2.3 Model

#### 2.3.1 From Short-Term Data to Long-Term History

We assume there that the repeated observations of a subject are sampled from a continuous function *t* ↦ *η_i_* (*t*) = (*η*_*i*1_(*t*), … , *η_iN_* (*t*)), where *η_ik_*(*t*) describes the decrease of cortical thickness of this *i*th individual at vertex *k*, such that

(1) $$\forall \;i\in \left\{1,\ldots ,I\right\}\;\forall \;j\in \left\{1,\ldots ,J_{i}\right\}\;\forall \;k\in \left\{1,\ldots ,K\right\}\;y_{\mathrm{ijk}}=\eta_{\mathrm{ik}}\left(t_{\mathrm{ij}}\right)+\epsilon_{\mathrm{ijk}},$$

where $\epsilon_{\mathrm{ijk}}∼N\left(0,\sigma^{2}\right)$ corresponds to the model noise, whose variance is *σ*^2^.

The function *t* ↦ *η_ik_*(*t*) describes the evolution of the time-series at node *k* for the individual *i*. Thus, the vector function *t* ↦ *η_i_* (*t*) = (*η*_*i*1_(*t*), … , *η_iN_* (*t*)) describes the continuous evolution on the graph for a particular individual, i.e., the spatiotemporal propagation of the signal over the whole brain. It corresponds to a spatiotemporal trajectory in the space of measurements. The trajectory *t* ↦ *η_i_* (*t*) is therefore able to reconstruct the existing observations (**y***_ij_*)_1 ≤ *j*≤*Ji*_, defined at the related time-points (*t_ij_*)_1 ≤ *j*≤*Ji*_, as shown on Figure 3B, but also generate an observation at any time t, potentially in the future.

The repeated data of each individual is a particular window in the long-term course of the disease that potentially overlaps with other patients. We aim to realign along a common time-line these short-term sequences by carefully analyzing the spatiotemporal patterns within each short-term snapshot. Nevertheless, to do so, we also need to account for the interindividual variability in cortical thickness measurements and trajectories of propagation across the network. The interindividual variability prevents us from considering any individual propagation as a good representation of the disease evolution.

Consequently, we assume that there exists a mean scenario of propagation, defined by a group-average spatiotemporal trajectory *t* ↦ γ _0_(*t*), represented on Figure 3C, such that each individual trajectory *t* ↦ *η_i_*(*t*) is a temporal and spatial variation of this mean scenario of changes, detailed in section 2.3.2. This typical scenario of change describes the mean pattern of spatiotemporal propagation of the signal and writes *γ*_0_(*t*) = (*γ*_1_(*t*), …,*γ_K_*(*t*)) where for all *k* ∈ {1,…, *K*}, *t* ↦ *γ_k_*(*t*) characterize the typical temporal evolution of the cortical thickness on the brain region related to the node *k*. As represented in Figure 2, each node has a different temporal profile of atrophy, accounting for the variation of the cortical thickness over time.

#### 2.3.2 Individual Estimation

Translating the generic framework introduced by Schiratti et al. (18) into this case requires to exhibit individual parameters that characterize the individual spatial and temporal variations to the mean, namely the *space shifting* and the *time reparametrization*.

##### 2.3.2.1 Time Reparametrization

We introduce a time-warp function *ψ_i_* (t) that corresponds to a time reparametrization that adjust the individual dynamics on a common time-line, which here is the average spatiotemporal trajectory *γ*_0_. For any patient *i* with observations (*y_ij_*)_1 ≤ *j* ≤ *Ji*_ at time-points (*t_ij_*)_1 ≤ *j* ≤ *Ji*_, *ψ_i_*(*t_ij_*) = *α*_i_(*t_ij_–t*_0_*–τ_i_*) + *t*_0_ where *t*_0_ is a common reference time of the reparametrization, *α_i_* encodes for the individual pace of propagation and *t*_0_ + *τ_i_* describes subject-specific time-shift to the mean disease onset. As such, if the acceleration factor *α*_i_ is greater than 1, it corresponds to a faster pace of cortical atrophy whereas *α*_i_ < 1 indicates a slower pace of atrophy. In the same way, the larger the value of the time-shift *τ_i_* is, the later the disease occurs. Therefore, it leads to write *η_i_*(*t*) = *γ*_0_(*ψ_i_*(*t*)) + ***ε***_*ij*_. It adjusts the pace at which the trajectory is followed for the *i*th individual.

##### 2.3.2.2 Space Shifting

In the space of measurements $R^{K}$, we consider individual observations and the mean trajectory *γ*_0_(*t*) as shown in Figure 3A. In order to account for the spatial variability of the individual trajectories, we assume that there exists, for any individual *i*, a vector *w_i_* ∈ $R^{K}$ called the space shift, that characterizes the spatial variations from *γ*_0_(*t*) to the observations as shown in Figure 3B. For any point on *γ*_0_(*t*), *γ*_0_(*t*) + *w*_i_ is assumed to be on the individual trajectory. Therefore, it is possible to translate all the points ${(\gamma_{0}(t))}_{t\;\epsilon \;R}$ to ${\left(\gamma_{0}+w_{i}\right)}_{t\;\epsilon \;R}$ as shown in Figure 3C. This collection defines the individual trajectory *η_i_*(*t*). This space shift must be orthogonal to the trajectory as it ensures the identifiability of the model. In fact, if the direction *w_i_* was not orthogonal to the trajectory, then the projection of *w_i_* on the geodesic *γ*_0_ would interfere with the individual time realignment induced by the dynamic parameters (*α_i_*,*τ_i_*).

Using mathematical tools from the Riemannian geometry beyond the scope of this study (26) shows that the *k*th coordinate of the individual spatiotemporal trajectory writes $\eta_{\mathrm{ik}}\left(t\right)=\gamma_{k}\left(\frac{w_{\mathrm{ik}}}{{\dot{\gamma }}_{k}\left(t_{0}\right)}+\psi_{i}\left(t\right)\right)$. As the space shift must be estimated in $R^{K}$, *w_i_* is supposed to be a linear combination of few independent components, in the spirit of independent component analysis (ICA) (27). It leads to consider *A* a *K* × *N_s_* matrix of *N_s_* independent directions, and (*s_ij_*)_1 ≤ *i* ≤ *I*, 1 ≤ *j* ≤ *Ns*_ parameters to estimate. $s_{i}=\left(s_{i1},\ldots ,s_{iN_{s}}\right)\in R^{N_{s}}$ correspond to parameters of individual *i* that characterize his spatial variations from the mean spatiotemporal trajectory. The orthogonality condition, mentionned in the previous paragraph, leads to consider a basis $\left(B_{1},\ldots ,B_{\left(K-1\right)N_{s}}\right)$ of matrices, whose columns are orthogonal to the direction of *γ*_0_(*t*), and parameters (*β_l_*)_1 ≤ *l* ≤ (*K*−1)*Ns*_ such that $A=\sum_{j=1}^{\left(K-1\right)N_{s}}\;\beta_{j}B_{j}$. This procedure allows to reduce the dimension of the parameters to estimate for each *w*_i_, from *K* to the chosen number of sources.

It leads to write:

(2) $$y_{\mathrm{ijk}}=\gamma_{k}\left(\frac{w_{\mathrm{ik}}}{{\dot{\gamma }}_{k}\left(t_{0}\right)}+\alpha_{i}\left(t_{\mathrm{ij}}-\tau_{i}-t_{0}\right)+t_{0}\right)+\varepsilon_{\mathrm{ijk}}.$$

#### 2.3.3 Curve Parametrization

In this paper, we consider a *straight line model* such that *γ_k_*(*t*) = *v_k_*(*t–t_k_*) + *p_k_*, *v_k_* accounting for the ratio of atrophy and *p_k_* for the thickness value at time *t_k_*. A linear decay in cortical atrophy is then represented by a straight line trajectory, parametrized by time, in the *K*-dimensional space as shown on Figure 3. Note that as shown on Figure 2, it is possible to parametrize the same curve with two distinct sets (*p*_1_, *t*_1_) and (*p*_2_, *t*_2_) preventing from having an identifiable model. We decided to fix the parameter *t_k_* among all the nodes such that for all *k* ∈{1,…,*K*} *t_k_* = *t*_0_, the time reference used in section 2.3.2.1, without any loss of generality as *t* ↦ *γ_k_*(*t*) is defined on R. Despite the linear form of each coordinate *t* ↦ *γ_k_*(*t*), the resulting model is non-linear as it includes among others, multiplication of individual and population parameters.

Finally, equation (2) becomes

(3) $$y_{\mathrm{ijk}}=p_{k}+w_{\mathrm{ik}}+v_{k}\alpha_{i}\left(t_{\mathrm{ij}}-\tau_{i}-t_{0}\right)+\varepsilon_{\mathrm{ijk}}.$$

This model therefore defines a distribution of multivariate straight line trajectories that accounts for the distribution of the individual trajectories.

#### 2.3.4 Spatial Smoothness

The model proposed in this paper deals with data that are spatially distributed on a graph *G* defined by a set of nodes $V=(x_{1},\ldots ,x_{K})$, where each node embeds a spatial coordinate in $R^{3}$. We expect a smoothly varying profile of atrophy across nodes. The proximity between edges is given by the distance matrix *D*.

In order to ensure small variations of the signal, we introduce a subset $V_{c}=\left(x_{d_{1}},\ldots ,x_{d_{N_{c}}}\right)\subset V$ whose vertices are called control nodes. Instead of estimating (*p_k_*)_1 ≤ *k* ≤ *K*_ (resp. (*v_k_*)_1 ≤ *k* ≤ *K*_) at all the nodes, we consider only the parameters at the control nodes ${\left(p_{d_{k}}\right)}_{1\leq k\leq N_{c}}$ (resp. ${\left(v_{d_{k}}\right)}_{1\leq k\leq N_{c}}$). We introduce a estimation function **x** ↦ *p*(**x**) (resp. **x** ↦ *v*(**x**)) for all x∈V such that, at the control nodes, the function is equal to the parameters: ∀*k* ∈ {1,…,*Nc*}, $p\left(x_{d_{k}}\right)=p^{d_{k}}$ (resp. $v\left(x_{d_{k}}\right)=v^{d_{k}}$). At the other nodes, the function is an interpolation of the parameter value at the control nodes weighted by the distance to each of them. Therefore, the control vertices control the evaluation of the parameters among all the nodes.

We choose a Gaussian kernel *K_b_* as interpolation splines: $\forall \;x,y\in V,\;K_{b}\left(x,y\right)=\text{exp}\left(-\frac{d{\left(x,y\right)}^{2}}{b^{2}}\right)$ where *d* is the geodesic distance on the mesh and *b* is the kernel bandwidth. This interpolation allows to remove the possible high frequencies, smoothing the signal spatially. Therefore, it leads to write:

(4) $$\forall x\in V,p\left(x\right)=\sum_{i=1}^{N_{c}}\beta_{p}^{i}K_{b}\left(x,x_{d_{i}}\right)\;\;\text{and}\;\forall x\in V,v\left(x\right)=\sum_{i=1}^{N_{c}}\;\beta_{v}^{i}K_{b}\left(x,x_{d_{i}}\right).$$

The parameters ${\left(\beta_{p}^{i}\right)}_{1\leq i\leq N_{c}}$ (resp.${\left(\beta_{v}^{i}\right)}_{1\leq i\leq N_{c}}$) are the solution of the linear system $p^{d_{k}}=\sum_{i=1}^{N_{c}}\;\beta_{p}^{i}K_{b}\left(x_{d_{k}},x_{d_{i}}\right)$ (resp. $v^{d_{k}}=\sum_{i=1}^{N_{c}}\;\beta_{v}^{i}K_{b}\left(x_{d_{k}},x_{d_{i}}\right)$).

Given these interpolations, equation (3) writes

(5) $$y_{\mathrm{ijk}}=p\left(x_{k}\right)+{\left(As_{i}\right)}_{k}+v\left(x_{k}\right)\alpha_{i}\left(t_{\mathrm{ij}}-\tau_{i}-t_{0}\right)+\varepsilon_{\mathrm{ijk}}.$$

Even though the distance computed for the cortical thickness corresponds to a distance on the brain cortex, it is possible to compute a connectivity distance based on the connectome, or even an appropriate combination of some of these distances. The challenging part is to put into correspondence areas defined by the connectivity matrices and other networks such as the FSAverage Atlas.

The choice of the set $V_{c}$ of control nodes among the whole set of nodes V is mostly determined by the choice of the bandwidth *b*: their uniform distribution is such that there is an approximate distance *b* between them. In the case of the cortical thickness, we have chosen a bandwidth equal to 16 mm which is representative of the spatial variability of the signal.

### 2.4 Algorithm

Equation (5) describes a mixed-effects model, introducing population and individual parameter in this high-dimensional non-linear model. We consider that (${\left(\alpha_{i}\right)}_{1\leq i\leq I}$, ${\left(\tau \right)}_{1\leq i\leq i}$, ${\left(s_{\mathrm{ij}}\right)}_{1\leq i\leq \mathrm{pI},1\leq j\leq N_{s}}$) are random-effects of the model, leading to write ∀ *i* ∈ {1,…, *I*} ∀*j* ∈ {1,…, *N_s_*}:

(6) $$\alpha_{i}=\exp\left(\xi_{i}\right)\;,\;\xi_{i}∼N\left(0,\sigma_{\xi }^{2}\right),\;\tau_{i}∼N\left(0,\sigma_{\tau }^{2}\right),\;\text{and},\;s_{\mathrm{ij}}∼\mathrm{Laplace}\left(0,\frac{1}{2}\right).$$

*α_i_* corresponds to the realization of a log-normal distribution so that it is always positive, preventing the individuals to present an increasing cortical thickness over time. Moreover, the Laplacian distribution of *s_ij_* arises from theoretical considerations as we need the model to be identifiable, i.e., the solution of the problem to be unique. Finally, these random-effects account for the statistical distribution of the individual trajectories. In the following, we consider **z** = ((*α_i_*)_1 ≤ *i* ≤ *I*_, (*τ*)_1 ≤ *i* ≤ *I*_, (*s_ij_*)_1 ≤ *i* ≤ *I*, 1 ≤ *i* ≤ *N_s_*_) as hidden variables.

Given equation (5) and the observations **y**, we would like to estimate the parameters $\theta =\left(t_{0},\;{\left(p^{d_{k}}\right)}_{1\leq k\leq N_{c}},\;{\left(v^{d_{k}}\right)}_{1\leq k\leq N_{c}},\;{\left(\beta_{k}\right)}_{1\leq k\leq N_{s}\left(K-1\right)},\;\sigma_{\tau },\;\sigma_{\xi },\;\sigma \right)$ as a maximum likelihood estimate (MLE) θ* = argmax *p*(**y**|***θ***). The natural way to perform such estimation in mixed-effects models is the Expectation-Maximization algorithm (28). Unfortunately, the E-step is intractable and it is not possible to sample according to the conditional distribution *p*(**z**|**y**, ***θ***). Therefore, we use a stochastic version of the EM algorithm coupled with a Monte-Carlo Markov-Chain method, namely, the Monte-Carlo Markov-Chain Stochastic Approximation Expectation-Maximization (MCMC-SAEM) algorithm that is able to deal with non-linear equations in a high-dimensional setting. The algorithm is proven to convergence (29) if the model belongs to the exponential family. In our case, it corresponds to consider that $p^{d_{k}}\;∼\;N\left(\bar{p},\sigma_{p}^{2}\right)$, $v^{d_{k}}\;∼\;N\left(\bar{v},\sigma_{v}^{2}\right)$ and $\beta_{k}\;∼\;N\left({\bar{\beta }}_{k},\sigma_{\beta }^{2}\right)$.

This leads to consider $z\;=\;\left({\left(\xi_{i}\right)}_{1\leq i\leq I},\;{\left(\tau_{i}\right)}_{1\leq i\leq I},\;{\left(s_{i}\right)}_{1\leq i\leq I},\;{\left(p^{d_{k}}\right)}_{1\leq k\leq N_{c}},\;{\left(v^{d_{k}}\right)}_{1\leq k\leq N_{c}},\;{\left(\beta_{k}\right)}_{1\leq k\leq N_{s}\left(K-1\right)}\right)$ as the extended hidden variables and $\theta \;=\;\left(t_{0},\;\bar{p},\;\bar{v},\;{\left(\beta_{k}\right)}_{1\leq k\leq N_{s}\left(K-1\right)},\sigma_{\xi },\sigma_{\tau },\sigma_{\bar{p}},\sigma_{\bar{v}},\sigma \right)$ as the parameters of the model. The latter introduces sufficient statistics *S* of the model that are functions of the observations **y** and latent variables **z**. The aim of such functions is to disentangle the maximization of the parameters *θ* and the simulation of the latent variables **z**.

The pseudo-code of the algorithm, reproduced in Algorithm 1, shows the different steps of the optimization until convergence. For further information about the steps of the algorithm, the reader is referred to Ref. (29–31) and references therein.

**Algorithm 1:** Estimation of the general and individual cortical thickness decrease with the MCMC-SAEM algorithm.

**Input**: Longitudinal dataset *y* = (*y_i,j_*)*_i, j_* of measurement maps, with the corresponding ages (*t_i,j_*)*_i, j_*.
Initial parameters *θ*^0^ and latent variables *z*^0^.
Geometrically decreasing sequence of step-sizes $\rho^{k}$.
Sufficient statistics $S^{k}$
*Initialization*: set *k* = 0 and *S*^0^ = *S*(*z*^0^).
**repeat**
*Simulation*: **foreach** block of latent variables *z_b_* **do**
Draw a candidate $z_{b}^{c}∼p_{b}\left(.|z_{b}^{k}\right)$.
Set $z^{c}=\left(z_{1}^{k\text{+1}},\ldots ,z_{b\text{-1}}^{k\text{+1}},\;z_{b}^{c},\;z_{b\text{+1}}^{k},\ldots ,z_{n_{b}}^{k}\right)$.
Compute the acceptation ratio $\omega =\text{min}\left[\text{1},\frac{q\left(z^{c}|y,\theta^{k}\right)}{q\left(z^{k}|y,\theta^{k}\right)}\right]$.
**end**
*Stochastic approx.*: $S^{k+1}\leftarrow S^{k}+\rho^{k}\left[S\left(Z^{k+1}\right)-S^{k}\right]$.
*Maximization*: $\theta^{k+1}\leftarrow \theta *\;\left(S^{k+1}\right)$.
*Increment*: set *k* ← *k* + 1.
**until** convergence;
**output**: Estimation of θ*.
Samples ($z^{s}$)^*s*^ approximately distributed following *q*(*z*|*y*,θ^∗^).

### 2.5 Simulation Study

**Figure 4:**
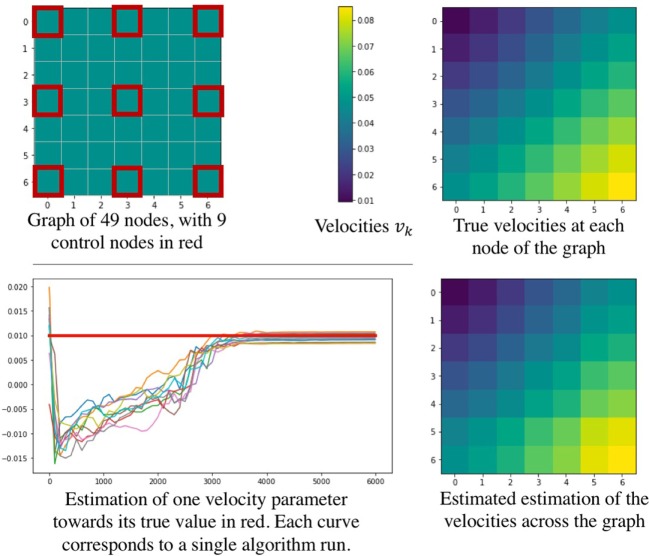
Simulation study performed to show the effectiveness of the parameter estimation procedure. The upper part describes the simulated graph (left) and the true velocities across the nodes (right). On the bottom part, a convergence example is given (left) as well as the estimated velocities estimated across the graph (right).

Since we introduce a new approach to deal with longitudinal data spatially distributed, we performed a simulation procedure to show both the legitimacy of the model used, and the effectiveness of the estimation procedure. To this end, we define a graph represented on the top left of Figure 4, representing a square mesh of 7 nodes per edge, thus 49 nodes in total. Among them, 9 equally distributed nodes represent the control nodes, in red on the figure. As we simulate data according to equation (3), we choose position and velocities across the node of the graph, as shown on the top right part of Figure 4. Then we simulated realizations (*ξ_i_,τ_i_*, (*s_ij_*)_1 ≤ *j* ≤ *N*_s__)_1 ≤ *i* ≤ *N*_ for 350 patients, from 4 to 12 visits each (2,980 visits in total) such that it represents 350 longitudinal trajectories of biomarkers spatially distributed. These data were used to find the parameters used to simulate them. Thus, we have performed 10 runs of the estimation procedure. In order to account for the stochasticity of the algorithm and the motion of the Markov Chains, the results in Table 1 are given with their standard deviation over 10 runs. As we need an initial value for the parameters, we initialized the algorithm without specific knowledge about the positions and velocities, contrary to the experience on the cortical atrophy, so it might reflect a worst-case scenario. Table 1 shows how well the algorithm performs on either control nodes or random nodes, as well as for the individual parameters.

**Table 1:** The table shows the ability of the algorithm to estimate the real value of the model parameters.

Parameter	Initial value	Final value	Real value	Error rate (%)
$p^{d_{\text{1}}}$	2.0	2.994 (±0.025)	3.0	0.2
$p^{d_{\text{20}}}$	2.0	3.663 (±0.146)	3.714	1.4
$p^{d_{\text{46}}}$	2.0	3.860 (±0.177)	3.9	1.0
$v^{d_{\text{4}}}$	1.0 × 10^−2^	2.84 (±0.24) × 10^−2^	3 × 10^−2^	5.3
$v^{d_{\text{21}}}$	1.0 × 10^−2^	5.86 (±0.49) × 10^−2^	6.25 × 10^−2^	6.2
$v^{d_{\text{41}}}$	1.0 × 10^−2^	7.83 (±0.65) × 10^−2^	7.8 × 10^−2^	0.4
*t*_0_	75	70.9 (±2.7)	70	1.3
$\sigma_{\tau }^{2}$	1.0 × 10^−3^	27.5 (±1.6)	25	10
$\sigma_{\xi }^{2}$	10^−7^	0.154 (±0.0008)	0.15	2.7
*σ*^2^	Not initialized	1.34 (±0.03) × 10^−5^	10^−5^	34

The bottom part of Figure 4 shows some results of the estimation procedure. On the left hand side, we provide an example of the stochastic estimations of a parameter over the iterations of the algorithm - the figure shows 10 independent runs. The right hand side presents the final estimation of the velocities across the node of the graph, showing that the model is likely to reproduce the real signal. Overall, these results confirm that such procedure seems reasonable to assess the validity of the model and of the estimation procedure in order to estimate the temporal profile of longitudinal data spatially distributed, such as the cortical atrophy.

## 3 Results

### 3.1 Initialization

We evaluated the propagation of the cortical atrophy thanks to cortical thickness values of 154 MCI converters (787 observations) distributed on a graph with 1,827 nodes.

**Figure 5:**
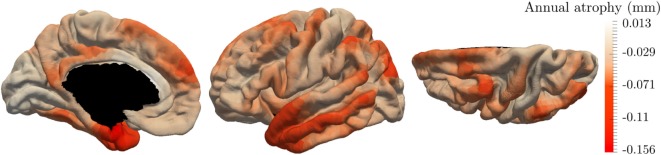
Annual rate of atrophy mapped over the brain surface used as initialization of our algorithm. Given one area, the corresponding rate of atrophy is obtained as the average regression coefficient of the linear regressions applied to each patient independently.

The initialization of the MCMC-SAEM algorithm requires initial values of the parameters ***θ*** and realizations **z**. We would like to draw attention on the realizations ((*α_i_*)_1 ≤ *i* ≤ *I*_, (*τ_i_*)_1 ≤ *i* ≤ *I*_, (*s_i_*)_1 ≤ *i* ≤ *I*_) and $\left({\left(p^{d_{k}}\right)}_{1\leq k\leq N_{c}},\;{\left(v^{d_{k}}\right)}_{1\leq k\leq N_{c}},\;t_{0}\right)$. The former are chosen equal to 0, leading to initial individual trajectories that are equal to the mean spatiotemporal trajectories. The pattern of atrophy is the same for everyone at the beginning. The latter variables, $\left({\left(p^{d_{k}}\right)}_{1\leq k\leq N_{c}},\;{\left(v^{d_{k}}\right)}_{1\leq k\leq N_{c}},\;t_{0}\right)$, are initialized based on the raw data. Besides *t*_0_ that is chosen as the mean age of the input observations, for each control node *k*, we computed linear regressions on the longitudinal thickness values of each patient. Then we average the regression coefficients, each corresponding to a given subject, such that we end up with one rate of atrophy *v_k_* per patch. Also, *p_k_* was chosen as the average thickness on a given patch. Figure 5 shows the map of the initial *v_k_* distributed over the cortical surface which looks reasonable.

The initializations of Figure 5 present areas with important cortical decrease over time, such as the temporal lobe and the hippocampus area. On the other hand, the primary visual cortex is less subject to a cortical atrophy. This initialization looks reasonable; however, these linear regressions are not able to reconstruct the individual observations, preventing from a characterization of personalized patterns of atrophy. It avoids describing the temporal and spatial variability of the individual propagations. Moreover, the linear regressions do not take into account the spatial coherence of the propagation as shown by the color-bar on Figure 5 where some areas present an important increase of the cortical thickness. It may be associated to the important noise within the data which is produced by the data acquisition, the extraction of the cortical thickness, and, the alignment on the same atlas.

Thanks to the model we introduced, we were able to reconstruct a mean (resp. individual) spatiotemporal trajectory, detailed in section 3.2 (resp. 3.3), that takes the form of the input measurements, preventing from working with percentiles or clusters that cannot be compared directly to the real observations. Due to the numerous number of hyperparameters and the stochastic behavior of the MCMC-SAEM, the algorithm was computed several times, each run of 100,000 iterations taking approximately 15 h. The runs led to similar results. In the following, the results are presented for the run that provided the best individual reconstruction, i.e., the smaller standard deviation *σ* of the noise. Its last estimation is of 0.29 mm, where 90% of the input data are between 1.5 and 4 mm.

### 3.2 Population Level

The model exhibits a long-term characteristic pattern of atrophy propagation from early MCI stage to post AD diagnosis. It corresponds to the group-average trajectory described in section 2.3.1 whose spatial (**w***_i_*) and temporal (*α_i_* and *τ_i_*) variations corresponds to individual spatiotemporal trajectories. It is important to mention that this trajectory is a mean trajectory in a statistical sense, as its parameters are the mean values of the individual parameters.

**Figure 6:**
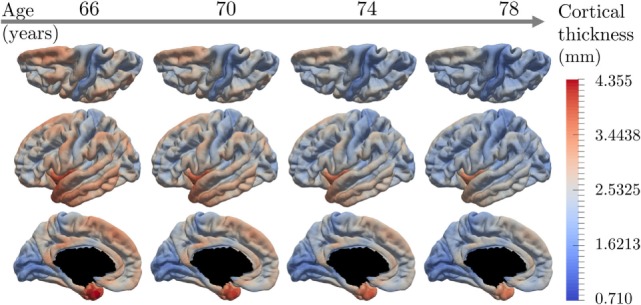
Estimated modes of evolution of the cortical thickness from 66 to 78 years old. This typical spatiotemporal pattern of atrophy propagation shows an important cortical loss in the superior frontal lobe, the temporal lobe, and the hippocampus region.

Figure 6 shows the temporal and spatial evolution of the cortical atrophy, from 66 to 78 years old. The brain medial and lateral views shows an important atrophy on the temporal lobe and the medial temporal lobe, especially the fusiform and the parahippocampical gyrus. An important cortical decrease is also discernible on the superior frontal gyrus and at the wider region defined by the inferior parietal lobe and the angular gyrus. On the other side, the prefrontal cortex, the primary visual cortex, the calcaris sulcus, and the post central gyrus are less subject to atrophy.

**Figure 7:**
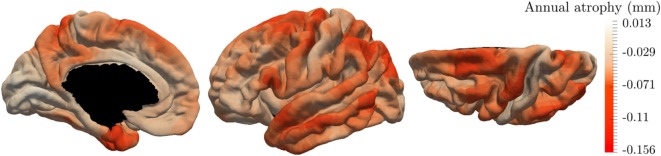
Final estimation of the annual rate of cortical loss observed during the typical pattern of atrophy propagation.

These results are supported by Figure 7 that shows the map of the annual atrophy *v_k_* for the mean spatiotemporal trajectory, distributed over the corresponding brain areas. The areas affected by the cortical atrophy correspond to previous knowledge (32–34) even tough the different measurements and methodologies lack in consensus. The patterns are still debated in order to find the best characterization of AD compared to normal aging or other neurodegenerative diseases. The proposed model may provide results for different populations on the same atlas, facilitating the comparison between diseases or with normal aging.

### 3.3 Individual Reconstruction

**Figure 8:**
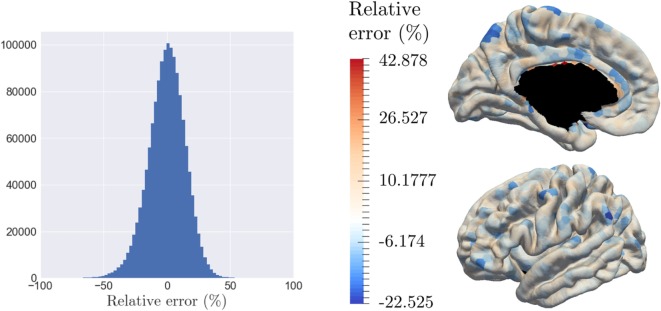
The model is able to reconstruct the data at the individual level, while smoothing the signal over the brain surface, with a relative error randomly distributed. **(A)** Histogram of the relative error of reconstruction of all individuals across all nodes. **(B)** Average relative error of reconstruction over each patch, distributed on the graph.

The model is able to characterize personalized patterns of atrophy propagation thanks to a reconstruction of the individual observations. The validation is assessed thanks to the relative error of reconstruction. As mentioned previously, the input data are noisy, at both a temporal and spatial level. As for the temporal part, the 154 patients represent 281.358 temporal profiles (time-series) over the 1,827 patches, from which only 6.4% present a monotonous profile of decrease. Given all the linear regression computed for the algorithm initialization, the mean (resp. the variance) of the corresponding R-square values is equal to 0.348 (resp. 0.307). On the other side, the spatial noise corresponds to high variation of the signal for neighbor nodes. Given this important noise, the goal of the reconstruction is not to reconstruct perfectly the data but rather to smooth the propagation over the brain and to capture individual tendencies of atrophy propagation. Thus, the 787 observations involve 1,437,849 reconstruction *y_ijk_*, whose relative error of reconstruction is represented on Figure 8A which confirms the hypothesis that the noise is a Gaussian distribution with a zero mean (*p*-value = 4.24.10^–109^ for a *t*-test comparison with a theoretical distribution of mean equal to zero). As highlighted by Figure 8B, that represents the relative error of reconstruction over the 1,827 patches, the error is mostly randomly distributed over the brain surface. It confirms that the reconstruction error does not have a spatial component as it is uniformly distributed over the brain surface. The color-bar was chosen according to the extreme values: it is important to mention that the larger error of reconstruction corresponds to areas that are close to the corpus callosum where the interpolation relies on a fewer number of control points.

**Figure 9:**
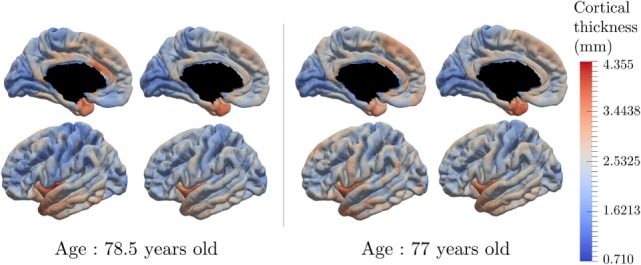
Real data and data reconstruction for subjects with a small space shift (right) and large space shift (left). The model is able to reconstruct the observed data, with a smoothing component, for subjects that present different characteristics.

Figure 9 presents the reconstruction of two different individuals who present various individual spatiotemporal trajectory, especially space shift norms that are either in the 10% bigger on the left hand side, or in the 10% smaller on the right hand side. The left part of each individual part corresponds to the input data whereas the right part is the corresponding reconstruction done by the model. It shows that the reconstruction is likely to represent the real data. The same color-bar was used as for Figure 6 to compare the individual data with the characteristic pattern of atrophy. Moreover, the spatiotemporal trajectory *η_i_* of individual *i* is not estimated only at the observed time-points but it is a continuous function of the time, as shown on Figure 3C. Therefore, it is possible to reconstruct the observation at any point, potentially in the future.

**Figure 10:**
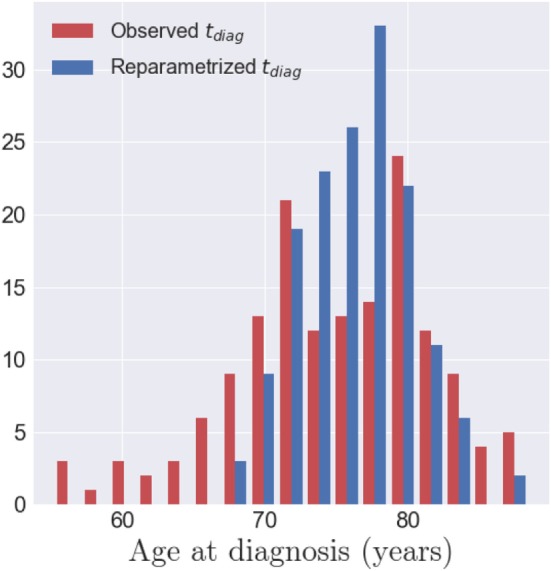
In red, the histogram of the observed age at diagnosis *t_diag,i_* for the 154 MCI converters. In blue, the histogram of the repatametrized age at diagnosis *ψ_i_*(*t_diag,i_*) once aligned on the common time-line. This shows that the age at diagnosis is mapped to a smaller range of time-points, in the model of cortical atrophy, suggesting that conversion to AD occurs at a specific stage of cortical atrophy.

One of the properties of the model is to exhibit individual temporal parameters, namely the acceleration factor *α_i_* and the time-shit *τ_i_*, which allow to reparametrize the individual dynamics on a common time-line. As the data used here correspond to the cortical thickness, the realignment is estimated thanks to structural biomarker dynamics. On the other side, the MCI converters have an age at disease onset, *t_diag_*, which corresponds to a clinical status. The latter is not straightforwardly related to the structural dynamics of the individual. In that sense, we decided to realign the age at onset *t_diag_*, a clinical biomarker, on the same time-line, assessed with the structural biomarkers. The observed age at diagnosis *t_diag,i_* are represented by the red histogram on Figure 10, which is not unimodal and present an important variance. The realignment of the clinical status is represented thanks to the distribution of (*ψ_i_*(*t_diag,i_*))_1 ≤ *i* ≤ *I*_, which is centered with a reduced variance. It suggests that the clinical conversion to AD, determined with *t_diag_* corresponds to a specific stage of the cortical atrophy.

**Figure 11:**
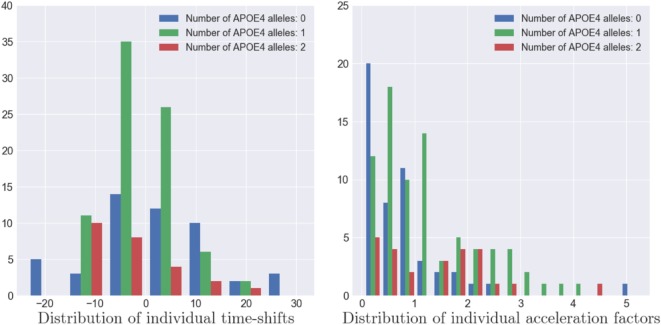
Distribution of the individual time-shifts (left) and the individual acceleration factors (right) for three types of APOE-*ϵ*4 population. A larger number of alleles of the APOE-*ϵ*4 genes is correlated to a faster pace of propagation of the Alzheimer’s disease (*p*-value ≃ 0.001) but not with an earlier atrophy onset (*p*-value ≃ 0.5).

As the model estimates individual spatiotemporal trajectories, it allows to describe the variability within the population. The distributions of (*α_i_*)_1 ≤ *i* ≤ *I*_, (*τ*)_1 ≤ *i* ≤ *I*_ and (**w***_i_*)_1 ≤ *i* ≤ *I*_ account for the distribution of the individual patterns of atrophy. Furthermore, the ADNI dataset provides, for each patient, multiple features, such as the number of alleles of the APOE-*ϵ*4 gene, the gender, the marital status, and the educational level. In the case of the APOE-*ϵ*4 gene, which is known as a genetic risk factor regarding AD (35, 36), we exhibited the distribution of (*α_i_*)_1 ≤ *i* ≤ *I*_ and (*τ_i_*)_1 ≤ *i* ≤ *I*_ for the subpopulations defined by the number of alleles of the gene as shown on Figure 11. The more alleles, the more likely to have AD (35, 37).

As shown on the left hand side of Figure 11, the patients with two alleles (resp. one allele) present a mean time-shift of −2.98 years (resp. −0.20 years) after the mean scenario, contrary to patient without APOE-*ϵ*4 alleles that present an average time-shift of 1.89 years, meaning that the more alleles, the earlier the atrophy onset occurs. However, we applied Mann–Whitney two-sided statistical tests that lead to insignificantly differences between the subpopulation. On the other side, same tests were conducted for the same subpopulation with the mean acceleration factor whose distributions are presented on the right hand side of Figure 11. In this case, the group of individual with no alleles presented an average acceleration factor of 0.780, statistically different from the group of individual with one alleles (resp. two alleles) that presented an average acceleration factor of 1.415 (resp. 1.236) with a *p*-value equal to 0.00104 (resp.0.00511). However, this acceleration factor is not statistically different between the population with one or two alleles (*p*-value = 0.51518), meaning that these subpopulation have similar rate of atrophy. Additional investigation on the gender, the marital status and the education level did not led to significant differences. It is important to mention that the Mann–Whitney test is sensitive to the number of samples whereas this study focuses on only 154 MCI patients that might lead to insignificant results in some cases, particularly in the case of the educational level (20 categories) or the marital status (unbalanced classes). Finally, it should mentioned that the tests conducted on the individual space shifts *w*_i_ and the related sources *s_i_* did not lead to significant results, mainly because these parameters account for the difference in brain size, and thus thickness, between people.

## 4 Discussion

The paper presents a mixed-effects model of the atrophy propagation that is able to characterize a typical pattern of propagation, and, that reconstructs individual observations and scenarios of atrophy. The model exhibits brain areas that are the most affected by the cortical atrophy, such as the parahippocampical gyrus, the temporal lobe, and the superior frontal gyrus. The lesions are less important in the primary visual cortex, the prefrontal cortex, and the primary sensomotory cortex. The model allows to account for the different temporal dynamics of the alterations that can be then compared and ordered.

The proposed model offers a wide versatility of instantiation in terms of profile of temporal variations (exponential decay, sigmoid decay) and spatial variations (resolution, number of control nodes, kernel bandwidth) as it defines a generic framework for the estimation of longitudinal signals spatially distributed. It should be compared to other types of graph-related approaches, such as supervoxels (38) or a vertex-cluster method (39). The latter has exhibited clusters of regression that show profiles of atrophy similar to our results. However, such models do not deal with individual characteristics neither directly with imaging data but rather with normalized values or percentiles, which restrict the interpretation. Further efforts should be concentrated on the validation and improvement of our model, possibly with more complex data and signal propagation.

The individual reconstructions also inform about subject-specific patterns of atrophy propagation, with potential personalized estimation of the cortical atrophy at future time-points. Further investigations have to be conducted to ensure the quality of the new observations the model is able to generate, so that one can exploit the outcome that the model can predict for an individual some years after his of her last visit. This should be done with a proper validation set to determine the population parameters, and a test-set to predict the individual parameters and thus the future observations. Consistent results might provide information about the structural biomarkers related to the progression of AD, such as in Ref. (40).

Another improvement of the model relies in the distance matrix computation. In this paper, the distance between the nodes is related to the distance on the brain surface, hiding potential effects of the neuronal connections. New distances might be computed based on functional connectivity or combination of different distances, in order to associate the functional and structural components of the brain that are supposed to be complementary in the disease process (41–43).

The model has the potential to exhibit the spatiotemporal propagation of any signal spatially distributed over a graph. It can be used in order to compare the patterns of propagation in distinct population, e.g., normal aging or any other neurodegenerative diseases. It is also a first step to define personalized patterns that would help for a future prognosis of the patient stages.

## Author Contributions

All authors listed have made substantial, direct and intellectual contribution to the work, and approved it for publication.

## Conflict of Interest Statement

The authors declare that the research was conducted in the absence of any commercial or financial relationships that could be construed as a potential conflict of interest.

## References

[1] Du AT Schuff N Amend D Laakso MP Hsu YY Jagust WJ Magnetic resonance imaging of the entorhinal cortex and hippocampus in mild cognitive impairment and Alzheimer’s disease. J Neurol Neurosurg Psychiatry (2001) 71(4):441–7. 10.1136/jnnp.71.4.441 11561025 PMC1763497

[2] Benzinger TL Blazey T Jack CR Koeppe RA Su Y Xiong C Regional variability of imaging biomarkers in autosomal dominant Alzheimer’s disease. Proc Natl Acad Sci U S A (2013) 110(47):E4502–9. 10.1073/pnas.1317918110 24194552 PMC3839740

[3] Amieva H Le Goff M Millet X Orgogozo JM Pérès K Barberger-Gateau P Prodromal Alzheimer’s disease: successive emergence of the clinical symptoms. Ann Neurol (2008) 64(5):492–8. 10.1002/ana.21509 19067364

[4] Wilson R Leurgans S Boyle P Bennett D. Cognitive decline in prodromal Alzheimer disease and mild cognitive impairment. Arch Neurol (2011) 68(3):351–6. 10.1001/archneurol.2011.31 21403020 PMC3100533

[5] Mura T Proust-Lima C Jacqmin-Gadda H Akbaraly TN Touchon J Dubois B Measuring cognitive change in subjects with prodromal Alzheimer’s disease. J Neurol Neurosurg Psychiatry (2014) 85(4):363–70. 10.1136/jnnp-2013-305078 23840054 PMC5225268

[6] Jack CR Knopman DS Jagust WJ Shaw LM Aisen PS Weiner MW Hypothetical model of dynamic biomarkers of the Alzheimer’s pathological cascade. Lancet Neurol (2010) 9(1):119–28. 10.1016/S1474-4422(09)70299-6 20083042 PMC2819840

[7] Fan Y Resnick M Wu X Davatzikos C Structural and functional biomarkers of prodromal Alzheimer’s disease: a high dimensional pattern classification study. Neuroimage (2008) 41(2):277–85. 10.1016/j.neuroimage.2008.02.043 18400519 PMC2682533

[8] Singh V Chertkow H Lerch JP Evans AC Dorr AE Kabani NJ Spatial patterns of cortical thinning in mild cognitive impairment and Alzheimer’s disease. Brain (2006) 129(11):2885 10.1093/brain/awl256 17008332

[9] Baron J Chételat G Desgranges B Perchey G Landeau B de la Sayette V In vivo mapping of gray matter loss with voxel-based morphometry in mild Alzheimer’s disease. Neuroimage (2001) 14(2):298–309. 10.1006/nimg.2001.0848 11467904

[10] Fonteijn HM Modat M Clarkson MJ Barnes J Lehmann M Hobbs NZ An event-based model for disease progression and its application in familial Alzheimer’s disease and huntington’s disease. Neuroimage (2012) 60(3):1880–9. 10.1016/j.neuroimage.2012.01.062 22281676

[11] Young AL Oxtoby NP Daga P Cash DM Fox NC Ourselin S A data-driven model of biomarker changes in sporadic Alzheimer’s disease. Brain (2014) 137(9):2564–77. 10.1093/brain/awu176 25012224 PMC4132648

[12] Young AL Oxtoby NP Huang J Marinescu RV Daga P Cash D Multiple orderings of events in disease progression. Information Processing in Medical Imaging. Isle of Skye, Scotland: Springer (2015). p. 711–22. 10.1007/978-3-319-19992-4_56 26223048

[13] Jedynak BM Lang A Liu B Katz E Zhang Y Wyman BT A computational neurodegenerative disease progression score: method and results with the Alzheimer’s disease neuroimaging initiative cohort. Neuroimage (2012) 63(3):1478–86. 10.1016/j.neuroimage.2012.07.059 22885136 PMC3472161

[14] Donohue M Jacqmin-Gadda H Goff ML Thomas R Raman R Gams A Estimating long-term multivariate progression from short-term data. Alzheimers Dement (2014) 10(5):400–10. 10.1016/j.jalz.2013.10.003 24656849 PMC4169767

[15] Gao S Hendrie H Hall K Hui S. The relationships between age, sex, and the incidence of dementia and Alzheimer disease: a meta-analysis. Arch Gen Psychiatry (1998) 55(9):809–15. 10.1001/archpsyc.55.9.809 9736007

[16] Devanand DP Pradhaban G Liu X Khandji A De Santi S Segal S Hippocampal and entorhinal atrophy in mild cognitive impairment: prediction of Alzheimer disease. Neurology (2007) 68(11):828–36. 10.1212/01.wnl.0000256697.20968.d7 17353470

[17] Bilgel M Prince JL Wong DF Resnick SM Jedynak BM. A multivariate nonlinear mixed effects model for longitudinal image analysis: application to amyloid imaging. Neuroimage (2016) 134:658–70. 10.1016/j.neuroimage.2016.04.001 27095307 PMC4912927

[18] Schiratti J-B Allassonnière S Colliot O Durrleman S Learning spatiotemporal trajectories from manifold-valued longitudinal data. In: Cortes C Lawrence ND Lee DD Sugiyama M Garnett R, editor. Advances in Neural Information Processing Systems. Curran Associates, Inc (2015). p. 2404–12.

[19] Durrleman S Pennec X Trouvé A Braga J Gerig G Ayache N. Toward a comprehensive framework for the spatiotemporal statistical analysis of longitudinal shape data. Int J Comput Vis (2013) 103(1):22–59. 10.1007/s11263-012-0592-x 23956495 PMC3744347

[20] Leuchter AF Newton TF Cook IA Walter DO Rosenberg-Thompson S Lachenbruch PA. Changes in brain functional connectivity in Alzheimer-type and multi-infarct dementia. Brain (1992) 115(5):1543–61. 10.1093/brain/115.5.1543 1422803

[21] Maguire EA Burgess N Donnett JG Frackowiak RSJ Frith CD O’Keefe J. Knowing where and getting there: a human navigation network. Science (1998) 280(5365):921–4. 10.1126/science.280.5365.921 9572740

[22] Broomhead DS Lowe D Radial Basis Functions, Multi-Variable Functional Interpolation and Adaptive Networks. Technical report. United Kingdom: Royal Signals and Radar Establishment Malvern (1988).

[23] Reuter M Schmandsky N Rosas H Fischl B. Within-subject template estimation for unbiased longitudinal image analysis. Neuroimage (2012) 61(4):1402–18. 10.1016/j.neuroimage.2012.02.084 22430496 PMC3389460

[24] Fischl B Sereno M Tootell R Dale A. High-resolution intersubject averaging and a coordinate system for the cortical surface. Human Brain Mapp (1999) 8:272–84. 10.1002/(SICI)1097-0193(1999)8:4<272::AID-HBM10>3.0.CO;2-4 10619420 PMC6873338

[25] Peyré G Péchaud M Keriven R Cohen LD Geodesic methods in computer vision and graphics. Found Trends Comp Graph Vis (2010) 5(3–4):197–397. 10.1561/0600000029

[26] Schiratti J-B Allassonniere S Colliot O Durrleman S A bayesian mixed-effects model to learn trajectories of changes from repeated manifold-valued observations. J Mach Learn Res (2017) 18(133):1–33.

[27] Allassonniere S Younes L A stochastic algorithm for probabilistic independent component analysis. Ann Appl Stat (2012) 6(1):125–60. 10.1214/11-AOAS499

[28] Dempster AP Laird NM Rubin DB Maximum likelihood from incomplete data via the em algorithm. J R Stat Soc Ser B (1977) 39:1–38.

[29] Allassonnière S Kuhn E Trouvé A Construction of bayesian deformable models via a stochastic approximation algorithm: a convergence study. Bernoulli (2010) 16(3):641–78. 10.3150/09-BEJ229

[30] Delyon B Lavielle M Moulines E Convergence of a stochastic approximation version of the em algorithm. Ann Stat (1999) 27:94–128.

[31] Kuhn E Lavielle M Maximum likelihood estimation in nonlinear mixed effects models. Comput Stat Data Anal (2005) 49(4):1020–38. 10.1016/j.csda.2004.07.002

[32] Whitwell JL Przybelski SA Weigand SD Knopman DS Boeve BF Petersen RC 3d maps from multiple mri illustrate changing atrophy patterns as subjects progress from mild cognitive impairment to Alzheimer’s disease. Brain (2007) 130(7):1777–86. 10.1093/brain/awm112 17533169 PMC2752411

[33] Jack CR Petersen RC Xu YC Waring SC O’brien PC Tangalos EG Medial temporal atrophy on mri in normal aging and very mild Alzheimer’s disease. Neurology (1997) 49(3):786–94. 10.1212/WNL.49.3.786 9305341 PMC2730601

[34] Scahill RI Schott JM Stevens JM Rossor MN Fox NC Mapping the evolution of regional atrophy in Alzheimer’s disease: unbiased analysis of fluid-registered serial mri. Proc Natl Acad Sci U S A (2002) 99(7):4703–7. 10.1073/pnas.052587399 11930016 PMC123711

[35] Strittmatter WJ Saunders AM Schmechel D Pericak-Vance M Enghild J Salvesen GS Apolipoprotein e: high-avidity binding to beta-amyloid and increased frequency of type 4 allele in late-onset familial Alzheimer disease. Proc Natl Acad Sci U S A (1993) 90(5):1977–81. 10.1073/pnas.90.5.1977 8446617 PMC46003

[36] Poirier J Bertrand P Kogan S Gauthier S Davignon J Bouthillier D Apolipoprotein e polymorphism and Alzheimer’s disease. Lancet (1993) 342(8873):697–9. 10.1016/0140-6736(93)91705-Q 8103819

[37] Corder E Saunders A Strittmatter W Schmechel D Gaskell P Small G Gene dose of apolipoprotein e type 4 allele and the risk of Alzheimer’s disease in late onset families. Science (1993) 261(5123):921–3. 10.1126/science.8346443 8346443

[38] Segovia F Górriz J Ramírez J Salas-Gonzalez D Álvarez I López M A comparative study of feature extraction methods for the diagnosis of Alzheimer’s disease using the adni database. Neurocomputing (2012) 75(1):64–71. 10.1016/j.neucom.2011.03.050

[39] Marinescu RV Eshaghi A Lorenzi M Young AL Oxtoby NP Garbarino S A vertex clustering model for disease progression: application to cortical thickness images. International Conference on Information Processing in Medical Imaging Boone, NC: Springer (2017). p. 134–45.

[40] Eskildsen SF Coupé P Fonov VS Pruessner JC Collins DL Initiative ADN Structural imaging biomarkers of Alzheimer’s disease: predicting disease progression. Neurobiol Aging (2015) 36:S23–31. 10.1016/j.neurobiolaging.2014.04.034 25260851

[41] Bullmore E Sporns O. Complex brain networks: graph theoretical analysis of structural and functional systems. Nat Rev Neurosci (2009) 10(3):186–98. 10.1038/nrn2618 19190637

[42] Damoiseaux JS Greicius MD. Greater than the sum of its parts: a review of studies combining structural connectivity and resting-state functional connectivity. Brain Struct Funct (2009) 213(6):525–33. 10.1007/s00429-009-0208-6 19565262

[43] Wee C-Y Yap P-T Zhang D Denny K Browndyke JN Potter GG Identification of mci individuals using structural and functional connectivity networks. Neuroimage (2012) 59(3):2045–56. 10.1016/j.neuroimage.2011.10.015 22019883 PMC3254811

